# Root plasticity: an effective selection technique for identification of drought tolerant maize (*Zea mays* L.) inbred lines

**DOI:** 10.1038/s41598-023-31523-w

**Published:** 2023-04-04

**Authors:** Wajhat-Un-Nisa, Surinder Sandhu, Rumesh Ranjan, Rakesh Sharda

**Affiliations:** 1 Present Address: Maize Section, Department of Plant Breeding and Genetics, Punjab Agricultural University, Ludhiana, 141004 India; 2 Department of Soil and Water Engineering, Punjab Agricultural University, Ludhiana, 141004 India

**Keywords:** Plant breeding, Climate sciences

## Abstract

The decline in tropical maize productivity due to climatic vulnerability is a matter of serious concern as being a food and feed/fodder commodity, it is an important crop for the sustenance of human life. Genetic selections and development of water deficit stress (WDS) tolerant commercial varieties have potential to offset the impact of changing temperatures and precipitation. For trait-specific genetic enhancement, there is a need to understand a suite of adaptation strategies for crop. We studied the response of various shoot and root traits in 71 maize inbreds of diverse origin under simulated sub-optimal water supply controlled conditions, delineated an array of traits which must be considered for selection for WDS and validated the inbreds harbouring tolerance to WDS for selection of authentic donor lines to develop WDS tolerant hybrids. A large data set was limited to uncorrelated traits based on principal component analysis and variability among maize lines was deciphered using heatmap dendrogram. We also reported the relevance of root anatomical plasticity to the inherent potential of lines to combat WDS. We recommend incorporating the changes in number and diameter of xylem and metaxylem under simulated controlled conditions as a part of precise phenotyping for WDS in maize. The study led to identification of WDS tolerant line LM22 in maize.

## Introduction

The major crop losses occur due to water deficit conditions and are predicted to get worse in future. This put an clarion call among plant breeders globally to develop crops’ that has the ability to withstand water deficit stress (WDS). In the context to South Asia, especially India, the worst drought was faced in the year 2002. It affected 60% of cropped areas affecting 85 million people (https://www.downtoearth.org.in/blog/drought-forever-44976). Generally, 42% of the land area in India is affected by drought. Most of the Indian states especially, North Eastern states, observed up to 40% drought during 2018–19 (https://www.business-standard.com). Due to climatic volatility, generally scattered rainfall during monsoon results in water logging at one place and drought in other. Due to decline in water table in most of the states of North India, crop diversification during *Kharif* from paddy to other crops like maize, pigeon pea, cotton etc. are being advocated. Among the myriad of cereals, maize offers multi-usage in form of grain and fodder along with speciality corn viz*;* green ear, baby corn, sweet corn, popcorn, and raw materials for industry.

Designated as the queen of cereals, maize surpasses all other cereals and food crops in its ability to adapt to diverse agro-climatic conditions, and being cultivated from 58° N to 55° S latitude. Concurrent climatic volatility has aggravated abiotic and biotic stresses, which have become a threat to maize yields. World maize yield and production are projected to decline by 15–20% per year due to heat and drought conditions^1^. Around the globe, maize is grown within 300–500 mm of precipitation, which does not suffice even at a critical level for normal grain yield^2^. In India, about 80% of maize in *Kharif* season is grown under rainfed conditions and faces the wrath of the erratic behaviour of rains^3^. This scenario calls for incorporating drought tolerance traits into maize germplasm to offset predicted yield losses and sustain maize productivity in vulnerable zones. The goal of drought tolerance is to maintain crop productivity during droughts^4^. This can be done in a number of ways, such as drought avoidance or desiccation prevention, possibly in combination by matching crop water use to water availability and recovery of growth after rewetting^5^. Several morpho-physiological properties, including root characteristics, are involved in the complex trait of drought tolerance^6,7^. The ability of plants to draw water from deep soil layers is made possible by a deep root system with thick roots and significant branching ability^8^. The root system is divided into two phases: embryonic roots and postembryonic shoot-borne roots. Embryonic roots include primary and seminal roots, which make up the majority of the seedling rootstock in the first few weeks after germination. Postembryonic shoot-borne roots include brace roots, crown roots, and roots that form underground (formed above ground). Another group of roots called laterals, which are also referred to as root tips, or hairs, play a pivotal role in nutrient absorption and water uptake and show the first line of response to any natural cue as any kind of drying effect or water stress leads to loss of their meristem^9^. These roots are typically very short^10^ and anatomically have an open late metaxylem (LMX) for most of their length^11^ which is responsible for their dominant role in water uptake^12^. According to the "stress gradient hypothesis” the fate of seedlings will affect the structure and dynamics of most plant populations^13^. As a result, phenotypic evaluation at the seedling stage is thought to be a desirable strategy since it is a high-throughput, low-cost method that saves space and time^14^. Therefore, to breed for water deficit stress in maize, it is imperative to conduct precise phenotyping of root traits and to visualize the anatomical changes in their architecture in response to moisture stress. Most of the researchers in past mainly concentrated their research work on the flowering date (anthesis and silking) and yield attributing traits to identify drought tolerant lines and generally ignore root traits which are tedious and time taxing in the field. It is challenging to maintain a uniform degree of drought stress over the field and to characterize root traits, as the root characteristics have been found very significant attributes for the survival of plants under any sort of abiotic stress^15^. Thus, hydroponic, being an effective system to study the plant responses under various abiotic stresses for root screening, is considered. This system provide uniform environment for growth condition till flowering stage by providing proper nutrient availability based on the crop. Being effective and rapid method of screening, many researchers around the world used this hydroponic system to decipher the variability of majority of traits in major crops at seedling stage viz., for nitrogen use efficiency in wheat^16,17^ drought in maize^18^, heat stress in sunflower^19^ and drought in rice^20^.

“Thinking locally and acting globally”; we hypothesied the experiment whether by the application of different concentration of osmolyte under hydroponic mimic drought environment and to screen the amount of variation for drought traits (root traits) in our available maize germplasm. Based on above hypothesis, this study aims to decipher the variability in root architecture of a core collection of diverse maize germplasm under controlled conditions; to simulate the effects of different concentrations of osmolyte-induced water deficiency on root and shoot architecture and to identify candidate drought-tolerant donor lines based on drought tolerance index (DTI). Efforts are made to dissect the anatomy of the root of inbreds, having the differential response to moisture stress, to unravel the changes in their vascular system using scanning electron microscopy and their relevance to WDS.

## Results

### Evaluation under hydroponics

**Figure 1:**
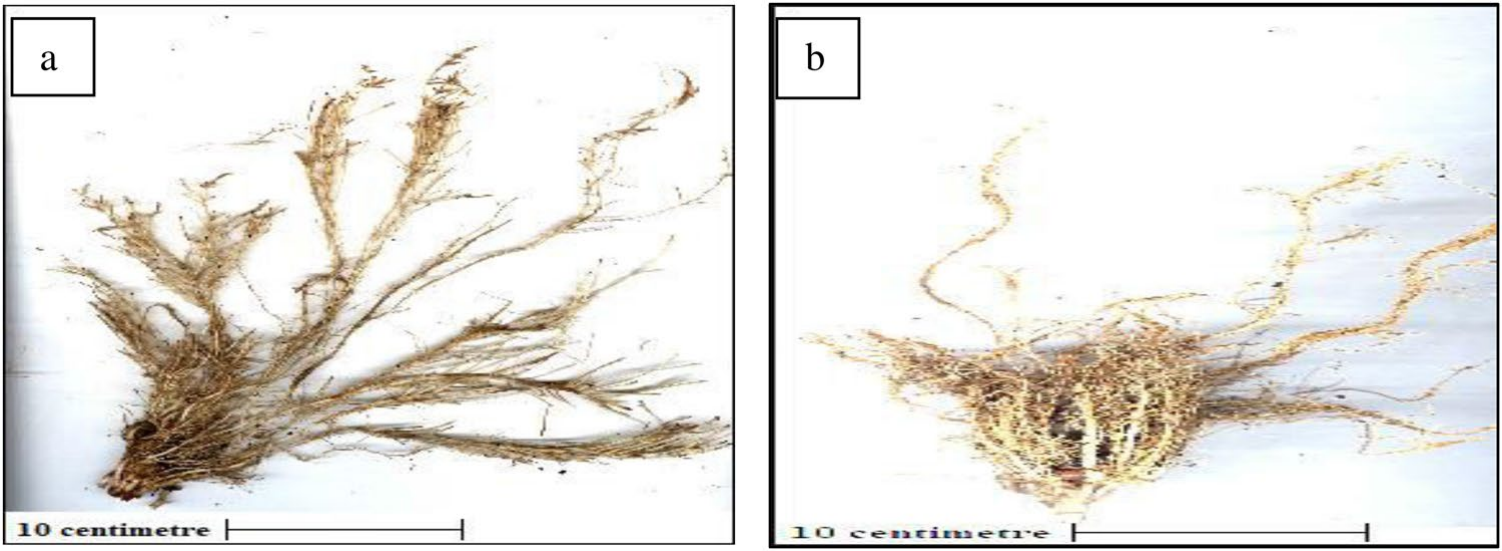
Root scanner images of maize inbred line LM22 under (**a**) control and (**b**) water deficit conditions.

**Figure 2:**
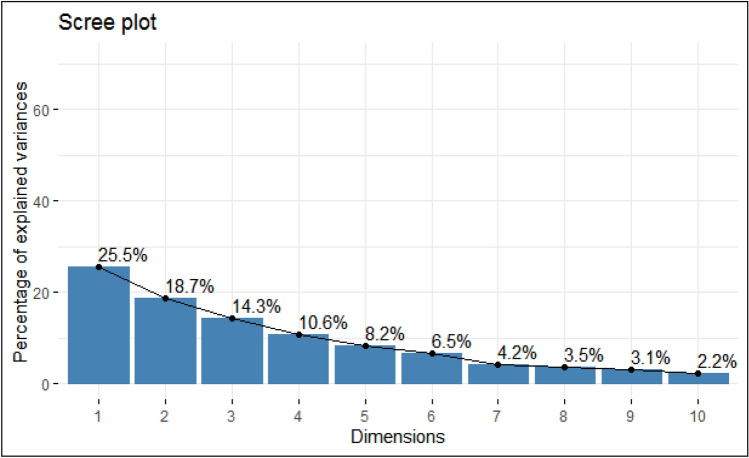
PCA predicting percentage of the variance depicted by first ten principal components.

Scan based precise root phenotyping was done through image acquisition representing diverse root architecture viz., primary, seminal, crown roots and laterals and data about RPA, TRL, RT, Forks and ARD (Fig. 1). Based on ANOVA for data recorded for seedling traits in 71 maize inbreds, a wide range of variability was observed under control (well-watered) and WDS (Table 1). The mean value of each trait was reduced under WDS in comparison to the control. Besides biomass traits, changes in chlorophyll (Chl) concentration and vigour (VIG) were recorded. The highest chlorophyll content (µmol/m) was recorded for PML 98 (19.55); LM22(18.5); PML296 (14.2); LM6 (13.7) and LM23 (13.15) whereas, PML 48, CML494, CML387 and CML444 recorded below 5 µmol/m. LM22 showed the highest vigour (4) followed by CML575, LM17, LM14, PML276 and LM15 (≅ 3.5) on the scale of 0–4. Based on PCA, the eigenvalue ranged from 3.3 for PC1 to 0.11 for PC13 (Table 2). The five PCAs, having eigenvalues greater than one, explained 77% of the cumulative variability. The variation explained by the first canonical vector was 25.5% followed by PC2 (18.7%), PC3 (14.3%), PC4 (10.2%) and PC5 (8.2%) (Fig. 2). The PC1 comprised FSW, FRW, DSW, VIG and CHL, PC2 comprised of VIG and TRL, PC3 comprised of forks and root tips, PC4 comprised ARD whereas PC5 comprised of DRW (Table 3).

**Table 1:** Descriptive statistics for seedling traits of 71 inbred lines under well-watered (control) and water deficit stress (WDS) conditions.

Trait	Mean standard deviation		Traits	Mean standard deviation	
	Control	Water deficit stress		Control	Water deficit stress
Fresh root weight (g)	20.3 ± 1.5	18.4 ± 3.2	No. of forks (numbers)	470.9 ± 10.2	424.9 ± 14.7
Fresh shoot weight (g)	39.4 ± 2.0	29.2 ± 3.1	Average root diameter (mm)	1.39 ± 0.2	1.01 ± 0.1
Dry root weight (g)	4.6 ± 1.0	2.8 ± 1.2	Root volume (cm^3^)	269 ± 10.9	226. ± 13.7
Dry shoot weight(g)	6.0 ± 0.2	4.4 ± 0.5	Maximum root length (cm)	34.0 ± 2.1	25.3 ± 3
Root projection area (cm^2^)	75.4 ± 1.4	67.7 ± 2.6	Vigour*	3.5 ± 0.3	2.5 + 0.8
Total root length (cm)	363.8 ± 4.3	317.4 ± 6.4	Chlorophyll content (µmol/m^2^)	8.1 ± 0.5	7.2 ± 1.4
No. of root tips (numbers)	434.3 ± 1.6	368.8 ± 4.6			

*g* fresh and dry root weight; shoot weight, *cm*^*2*^ root projection area, *cm* maximum and total root length (covering all root branches), *cm*^*3*^ root volume, *µmol/m*^*2*^ Chlorophyll content, number includes all root tips, hairs and forks (extending from main branch as seminals), *mm* average root diameter () indicate unit of respective trait.

*Vigour (0–4 scale): 0 (representing dead), 1 (very poor), 2 (poor health), 3 (reduced vigour but with browning of leaf tips) 4 (deep green leaves with no wilting or chlorosis: high vigour).

**Table 2:** Eigen values of first five principal components for seedling traits in 71 maize inbred lines under water deficit condition.

	PC1	PC2	PC3	PC4	PC5
Eigen values	**3.31**	**2.43**	**1.86**	**1.38**	**1.06**
Percent variance	25.5	18.7	14.3	10.6	82
Cumulative variance	25.5	44.2	58.5	69.1	77.3

Significant values are in [bold].

**Table 3:** Trait contribution towards principal component for root and shoot traits in 71 maize inbreds under water deficit condition.

Trait	PC1	PC2	PC3	PC4	PC5
Fresh root weight (g)	**0.473**	0.129	− 0.071	− 0.059	− 0.010
Fresh shoot weight (g)	**0.471**	0.108	− 0.020	− 0.062	− 0.118
Dry root weight (g)	0.059	0.025	0.112	0.020	**0.878**
Dry shoot weight (g)	**0.380**	0.050	0.185	− 0.088	− 0.054
Root projection area (cm^2^)	**0.388**	0.151	− 0.143	− 0.087	− 0.129
Total root length (cm)	**0.421**	0.067	0.036	0.037	0.181
Maximum root length (cm)	0.045	− **0.350**	− **0.444**	− **0.417**	0.048
No. of forks (numbers)	0.025	**0.488**	− **0.287**	− 0.326	− 0.024
Average root diameter (mm)	0.128	− **0.470**	**0.349**	0.088	− 0.196
Root volume (cm^3^)	0.168	− **0.415**	**0.380**	0.191	− 0.093
Number of root tips (RT)	0.160	− 0.211	− **0.488**	**0.377**	0.187
Vigour*	0.041	− 0.096	− **0.292**	**0.313**	− 0.108
Chlorophyll content (µmol/m^2^)	0.064	− **0.364**	0.237	0.020	0.257

Significant values are in [bold].

**Figure 3:**
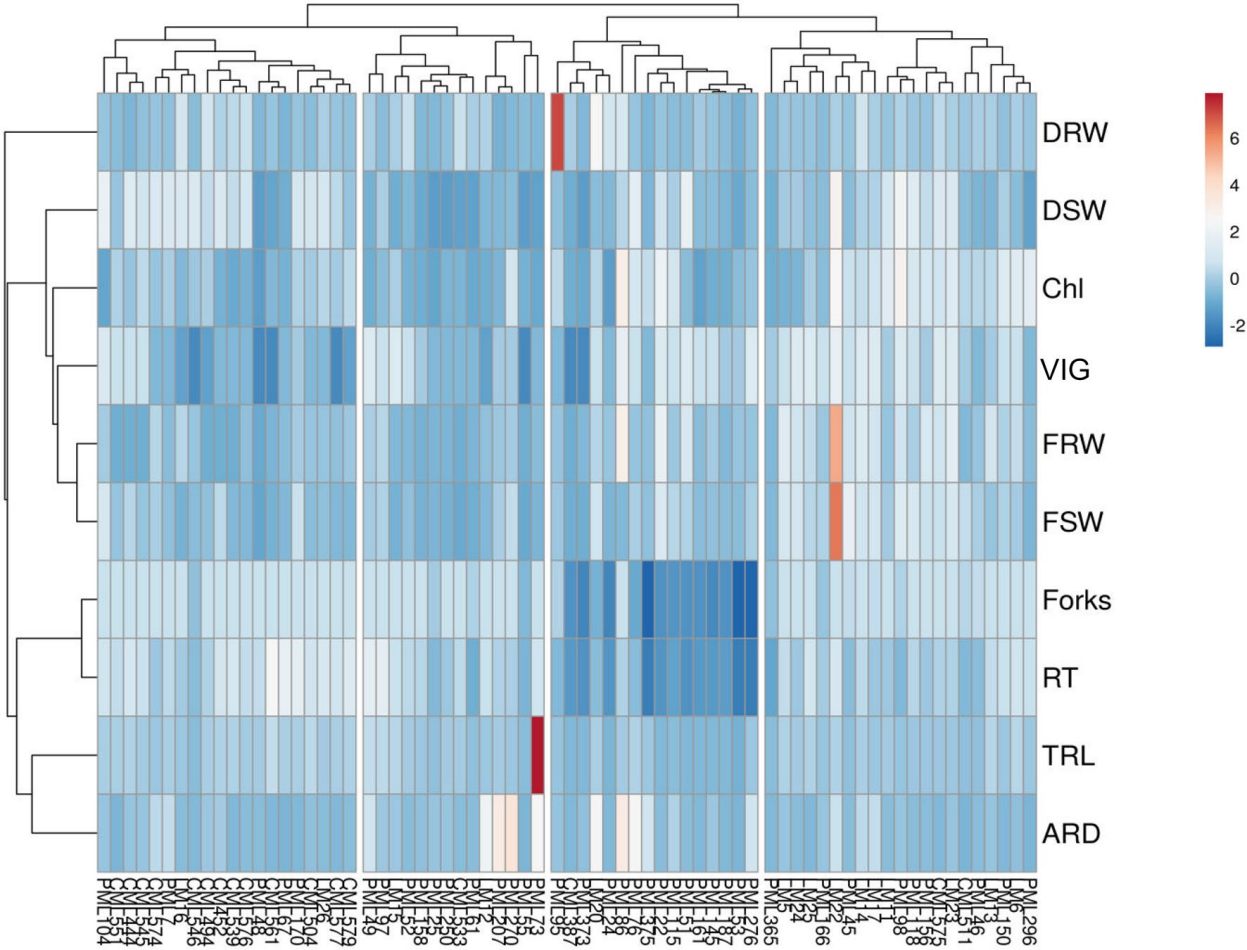
Heatmap depicting four clusters for 71 inbreds based on ten seedling traits. The colour code from darker blue to darker red represents the lower to higher values for root and shoot traits under water deficit condition.

PCA delineated 10 out of 13 traits for effective selection for WDS in maize, viz*.,* FRW, FSW, DRW, DSW, Chlorophyll, Vigour, TRL, RT, forks, and ARD. For further analysis, these 10 traits have been used. The multivariate analysis, conducted for 10 seedling traits under osmolyte-induced stress, led the grouping of 71 inbreds in four distinct clusters. Cluster means are given in Table 4. cluster I recorded the highest mean values for DSW, TRL, RT and Forks whereas, the clusters II and III showed intermediate mean values for all traits. Cluster IV recorded the highest means for fresh root weight (FRW), fresh shoot weight (FSW), dry root weight (DRW), dry shoot weight (DSW), vigour (VIG) and chlorophyll content (Chl). The relative performance for seedling traits under WDS was depicted through Heatmap cluster analysis. It showed two types of dendrograms: a genotype dendrogram with a vertical position and a character dendrogram with a horizontal position (Fig. 3). The intensity of colour corresponds to the value for each trait. The genotypes depicting darker red colour represent the higher value of the trait and are considered as tolerant to WDS, whereas, the darker blue represents lower values which are taken as susceptible to WDS. As deciphered in the heatmap dendrogram (Fig. 3), inbreds were grouped into four distinct clusters and traits into two. The FRW, FSW, DRW, DSW, VIG and Chl content were clustered into one group whereas root traits viz. RT, Forks, TRL and ARD were clustered in other. PML 73 exhibited the highest value for TRL and PML 95 for DRW. LM 22 exhibited the highest intensity for biomass traits viz., FSW and FRW. For other traits viz*.,* DSW, VIG, Chl content and root length, LM 22 inferred high values. The pictorial depiction can be utilised to identify donor lines for each trait.

**Table 4:** Cluster means for seedling traits in maize stock of 71 inbreds under water deficient stress condition.

Clusters/traits©	FRW	FSW	VIG	Chl	DRW	DSW	TRL	RT	Forks	ARD
Cluster I	10.72	29.24	2.04	6.24	2.25	8.01	506.25	486.75	494.5	0.58
Cluster II	10.82	21.83	2.26	4.05	2.09	2.17	332.67	407.61	479.07	1.85
Cluster III	21.59	32.31	2.65	7.26	4.24	5.27	239.21	162.18	211.93	1.37
Cluster1V	31.08	60.58	2.95	9.77	2.67	7.07	404.46	376.77	484.63	0.63

*©FRW (g)* fresh root weight, *FSW (g)* fresh shoot weight, *VIG* vigour, *Chl (µmol/m*^*2*^*)* chlorophyll content, *DRW (g)* dry root weight, *DSW (g)* dry shoot weight, *TRL (cm)* total root length, *RT* number of root tips, *ARD (mm)* average root diameter.

DTI, well documented as a reliable criterion to identify WDS^20^, was used to identify tolerant donor lines. DTI ranged from 0.01(CML533)-14.6 (LM22) for FRW; 0.02(PML55)-18.6(LM22) for FSW; 0.01(PML 73)-11(PML95) for DRW; 0.01 (CML533)-8.5(LM22) for DSW; 0.07(PML53)-7.62 (PML 73) for TRL; 0.07 (PML276) -3.7 (LM22) for RT and 0.2 (PML 296)-18 (PM207) for ARD. Inbreds were ranked from highly tolerant (high DTI) to highly susceptible (low DTI). Based on DTI ranks for seedling traits, inbreds with the extreme response for each trait are depicted in Table 5. LM22 showed the highest DTI for FSW, FRW, DSW, RT and ARD followed by CML 575 present in the tolerant group having higher values for FSW, FRW, DRW and DSW followed by CML 574, PML 98 and LM26. The cumulative DTI for root and shoot traits demarcated WDS tolerant lines (having higher values of DTI for five traits). A core set of 20, out of 71, inbreds was constituted representing differential DTI for various shoot and root traits.

**Table 5:** Trait based drought tolerant index (DTI) ranking of top twenty inbred lines of maize.

Rank	Inbred /trait©	FRW-DTI	Inbred/trait	FSW-DTI	Inbred/trait	TRL-DTI	Inbred/trait	RT-DTI	Inbred/trait	ARD-DTI	Inbred/trait	DRW-DTI	Inbred/trait	DSW-DTI
1	LM22	14.6	LM22	18.6	PML73	7.62	LM22	3.7	PML207	18.20	PML95	11.21	LM22	8.5
2	LM14	7.10	LM24	5.84	LM22	3.71	LM26	2.84	PML86	15.22	LM22	8.378	CM452	5.19
3	CML575	6.21	PML98	5.05	LM6	2.87	CML561	2.65	PML76	10.71	PML24	2.561	PML104	4.66
4	LM5	5.87	PML45	4.80	LM20	2.52	PML49	2.49	LM12	7.52	LM20	1.837	LM16	3.97
5	PML156	5.11	LM23	3.82	CML561	2.50	LM15	2.19	PML270	7.47	LM14	1.501	LM23	3.87
6	PML22	4.16	CML575	3.06	LM15	2.41	CML577	2.08	LM20	7.22	LM12	1.179	CML504	3.53
7	LM24	4.13	PML22	2.82	PML49	2.16	LM5	2.02	LM14	6.98	CML579	0.974	LM11	3.52
8	LM16	3.53	PML156	2.78	CM452	2.11	CM452	1.89	LM22	6.37	CML533	0.958	CML574	2.99
9	PML98	3.33	PML170	2.52	CML539	2.02	LM12	1.89	LM17	6.32	PML76	0.838	PML7	2.75
10	LM6	1.31	LM17	2.43	PML76	1.91	LM23	1.79	PML73	4.89	PML52	0.764	PML98	2.56
11	PML45	2.88	PML270	2.24	PML104	1.91	PML104	1.78	PML375	4.60	LM23	0.718	CML575	2.53
12	LM23	2.82	CML511	1.91	LM5	1.90	CML539	1.73	CML574	3.95	CML575	0.676	PML170	2.5
13	PML97	2.19	LM20	1.86	LM11	1.75	PML270	1.7	PML276	3.84	PML86	0.622	CML577	2.41
14	LM13	2.07	PML166	1.85	CML577	1.70	PML86	1.69	CM452	3.18	CML576	0.574	PML115	2.35
15	LM17	1.83	LM25	1.73	LM24	1.61	PML170	1.68	PML7	2.99	LM16	0.563	PML97	2.04
16	LM12	1.38	PML118	1.68	PML86	1.57	LM11	1.63	LM24	2.89	LM15	0.531	PML156	2.01
17	LM25	1.37	PML276	1.63	CML576	1.57	PML76	1.59	LM6	2.84	CML387	0.484	LM24	1.98
18	PML86	1.2	LM6	1.55	PML48	1.51	LM20	1.58	PML145	2.68	CML561	0.479	LM6	1.88
19	PML150	1.19	PML97	1.36	LM12	1.45	PML48	1.54	PML49	2.54	PML48	0.477	LM26	1.85
20	PML51	1.18	CML444	1.30	LM16	1.39	PML166	1.52	CML561	2.49	CML539	0.475	CML579	1.66

*©FRW (g)* fresh root weight, *FSW (g)* fresh shoot weight, *DRW (g)* dry root weight, *DSW (g)* dry shoot weight, *TRL (cm)* total root length, *RT* number of root tips, *ARD (mm)* average root diameter.

### Validation of the core set under variable osmolyte content

**Figure 4:**
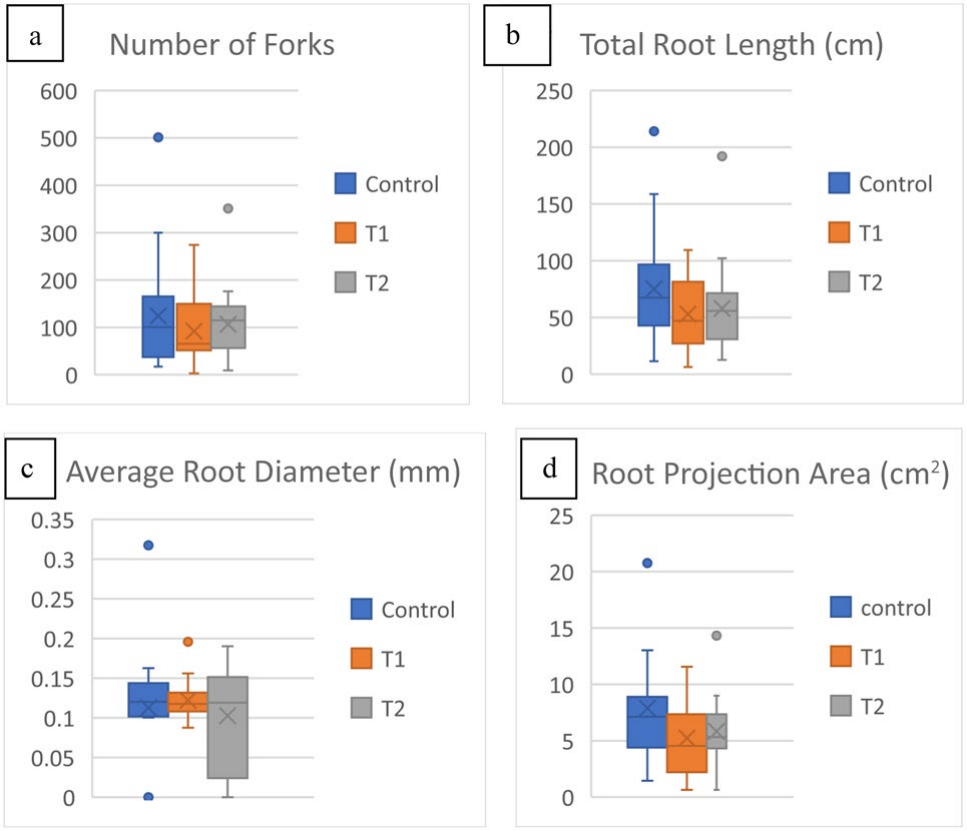
Box and Whisker charts showing variation in root morph traits measured for control, (T1) PEG 15% PEG and T2 (20%) treatments (**a**) forks, (**b**) total root length (TRL)-cm, (**c**) average root diameter (ARD)-mm, (**d**) root projection area (RPA)-cm^2^.

The constituted core set was selected to validate their response to WDS using different concentrations of osmolyte. The concentration of osmolyte was increased (from 10% in experiment 1) to 15% (T1) and 20% (T2) in the pot experiment under controlled conditions to authenticate the selection of WDS tolerant lines. Significant differences were observed among 20 inbreds in terms of data recorded for root and shoot traits at higher concentrations of osmolyte (Table 6). The mean maximum root length (MRL) increased in both T1 and T2 for tolerant lines whereas, the shoot length (SL) decreased with increased concentration of osmolyte ranging from 5.5 to 23 cm in T1 and from 3.5 to 18.8 cm in T2). Box and whisker charts (Fig. 4) showed the variation for root morphological traits. The mean RPA decreased with increasing concentration of osmolyte. TRL successively decreased with increasing concentration of osmolyte. However, a significant increase has pertained to the number of root tips in T1. The increase in the number of root segments was slightly higher in T1 than T2. The number of forks was reduced gradually from T1 to T2.

**Table 6:** Analysis of variance for seedling traits under control and variable WDS condition.

Trait	Mean sum of squares			Range			Mean		
	Control	T1	T2	Control	T1	T2	Control	T1	T2
Max root length (cm)	*	***	***	10.5–23	6.75–25	13.5–31.5	18	25	16.56
Max shoot length (cm)	*	***	***	19–42	5.5–2.6	3.5–18.75	27.525	13.87	11.17
Fresh root weight (g)	***	***	***	0.26–2.05	0.01–0.2	0.03–1.65	0.793	0.49	0.52
Fresh shoot weight (g)	***	***	***	0.31–2.01	0.03–1.33	0.01–0.56	0.897	0.43	0.20
Dry shoot weight (g)	*	***	***	0.01–0.2	0.01–0.2	0.01–0.22	0.09	0.09	0.02
Dry root weight (g)	***	***	***	0.26–2.05	0.01–0.20	0.01–0.10	0.09	0.09	0.11

T1: 15% PEG6000 and T2: 20% PEG6000.

*Significant at 5% level of significance; ***significance at 1% level of significance.

### Scanning electron microscopy (SEM)

**Figure 5:**
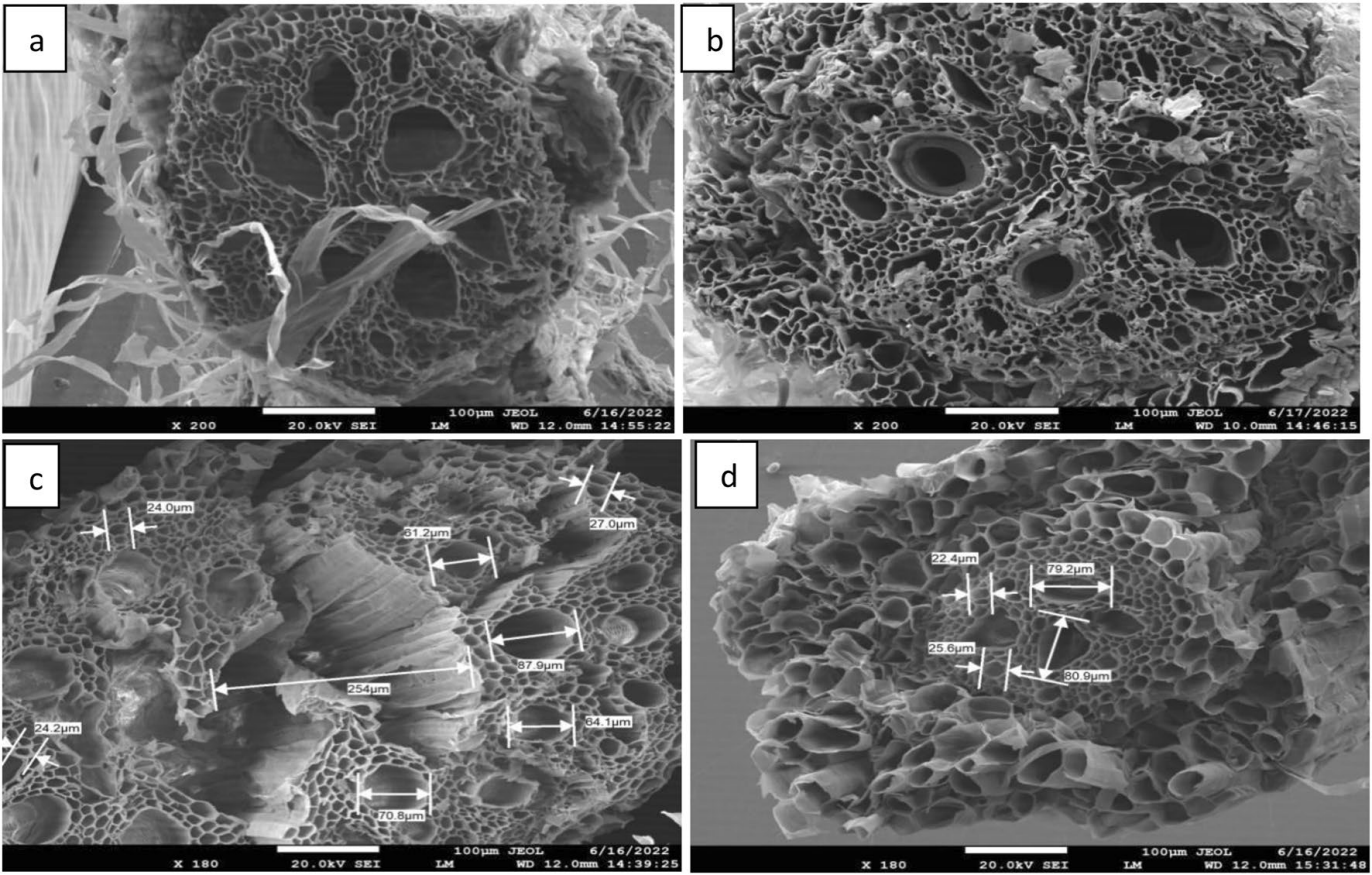
Scanning electron microscopy, (**a**) cross section of root under control (LM22) at magnification of ×200 and scale bar 100 µm showing meta xylem and xylem of larger diameter and visible aerenchyma, (**b**) root of the treated plant with PEG 6000 at magnification of ×200 and scale bar 100 µm showing decrease in the size of vessels. (**c**) Cross section area of root under control (CML494) at ×180 and scale bar 100 µm showing larger diameter of meta xylem and xylem, (**d**) root of the treated plant (PEG 6000) representing higher reduction in number of meta xylem and xylem along with diameter.

To validate the experiment results, the most tolerant maize inbred line LM22 and most susceptible CML494 (based on DTI) were selected for anatomical studies under WDS conditions using 20% osmolyte and control (well-watered) for comparison. The experiment was performed on samples of 20 days old primary roots. SEM revealed anatomical alternations in secondary vessels in response to WDS. Tolerant and susceptible lines differed remarkably in their response to WDS in terms of diameter and number of metaxylem and xylem. At 200× magnification, LM22 exhibited higher numbers of xylem and meta xylem and the respective diameter of meta xylem vessels were larger in comparison to those in CML 494 under control (Fig. 5). In the case of osmolyte-treated root scans, LM 22 maintained the number of secondary vessels with a slight decrease in their diameter. The scanning of CML494 (at magnification a of 180×) roots under control and WDS deciphered a high reduction in both the number and diameter of meta xylem and xylem under WDS in comparison to control. The reduction in the number of secondary vessels was visualised through the images (Fig. 5). The average diameter of secondary vessels in LM 22 was 85.9 µm whereas 76.7 µm in CML 494 under WDS. It differentiated the response of tolerant and susceptible lines to WDS. Moreover, at higher magnification, the walls of the vessels were not suberized after osmolyte treatment which demonstrates that the reduction in diameter of the secondary vessels can be authentically relied on the reduction in root diameter and not due to any effect of osmolyte.

## Discussion

Water stress due to drought is probably the most significant abiotic factor limiting plant growth and development. Reducing cell division, inhibiting cell elongation, slowing down photosynthetic activity, and altering root anatomy are only a few of the activities that are impacted by WDS in plants^21^. Water shortages during mid to late vegetative development and flowering have a significant impact on reproductive tissues, root morphology, and ultimate grain yield in maize. Most of the researchers in past focused on above-ground specific traits for the improvement of maize germplasm under WD stress whereas below-ground root traits were avoided due to hectic and time-consuming phenotyping. The root is the main organ through which plants absorb water and nutrient from the soil and is also the first organ sensitive to WDS^22^. Under unfavourable external conditions, the root system enables a variety of adaptive responses at the cellular and organ level and ensures a high level of plasticity^23^. Due to the hidden nature of the root structure in the soil and the high complexity of the root system, in situ root phenotyping has lagged. Conventional root phenotypic methods such as shovelomics, trench and mesh bags were highly labour oriented and time-consuming. Therefore, it has been necessary to create quicker and more precise techniques for in-situ observation of root plasticity under WDS. To circumvent these constraints, we used a simple and affordable method of hydroponic system with minimum impedance and soil strength variations, providing uniform moisture, and easy extraction of intact roots. Osmolyte-driven Polyethylene glycol (PEG) is the best solute for imposing low water stress that is reflective of the type of stress imposed by drying soil^24^ as these molecules generally do not enter the root and mimic the drought in a regulated manner. PEG treatment can well simulate a drought environment because it lowers the external free water concentration without changing the ionic makeup of the cell and lowers leaf water potentials^25^. So, by restricting access to water, it is possible to evaluate how the root system reacts to drought stress in solution cultures without causing root damage. Hydroponic screening with high molecular weight PEG osmolyte to study the root traits under drought at the seedling stage in maize has been advocated by many authors^26^. As for root phenotyping is concerned manual method has always been laborious and less precise therefore, root imaging was used in this study to authenticate the results. Because of its low cost and high efficiency, the image-based analysis provides more possibilities for high-throughput root phenotyping. It has been widely used in root phenotyping platforms in a wide variety of root growth systems, including soil-filled^27^, hydroponic/semi-hydroponic^28^ and gel/agar-based systems^29^.

Maize lines with various genetic origins and backgrounds revealed various levels of drought resistance and variable root architecture features at the seedling stage when grown in water-stressed settings. The significance of ANOVA suggests that inbred lines under consideration behave differently for root traits under WDS and Control which implies that sufficient variation for root traits is present in our studied lines and there is scope for genetic improvement for WDS. The effectiveness of breeding efforts depends on identifying maize germplasm with improved stress tolerance features and searching for drought-tolerant maize lines. Similar results are reported^30^. Our data also showed an increase in root length than shoot length with an increase in PEG concentration compared to the control. Maize responds to WDS by re-directing root growth and dry matter accumulation away from the shoot to root^31^. Increased concentrations of xyloglucan endo transglycosylase, transglycosylase/hydrolases, and other cell wall-loosening enzymes at the root tip are responsible for this transition, which entails an increase in root cell wall extensibility as a result the root grows steadily due to these modifications^32^.

The root mass and rooting depth increases the plant’s ability to cope with drought stress^33,34^. Hence, such traits are also considered important for identifying potential parents for hybrid development^35^. Plasticity in root morphological traits such as root angles, rooting depth, root diameter, the number of root branches and length of root hairs along with anatomical traits like number and diameter of metaxylem, xylem, aerenchyma and cortical cell file number determine the adaption for improved water use efficiency in crop species^36^. The present study revealed that water stress significantly affects maize growth processes resulting in a decrease in total root length, root biomass, root volume, shoot length and shoot biomass. A Significant decrease in root and shoot parameters under water stress conditions has been reported earlier^37^. This was in agreement with the results obtained in rice^38^,where the shoot heights of all the irrigated genotypes were higher than their corresponding stressed plants. The findings also agreed with the reports of higher shoot heights for the irrigated site than the rain-fed for 140 maize full-sib families tested for their tolerance to drought in Florida^39^. Our results also reflect that DSW were mostly affected under WDS compared to control where DRW show considerable changes. This might be due to DRW is generally contributed by RPA, TRV and TRL^40^.

The principal component analysis or canonical root analysis was used to analyse the interrelationship among a large set of variables. The first five components were explaining 77% of total variations. The differential behaviours of inbreds in terms of colour intensity for test traits were reflected through heatmap dendrogram. Heatmap is the graphical representation of data values in a colour code scheme. Both root and shoot traits showed variable colour schemes however, DSW, number of forks, RT, ARD, Chl, VIG showed higher positive values in maximum genotypes. Differential response of various physiological and biochemical traits for drought through heat map was reported in wheat^41^.

Water deficit inhibits the growth of plants by inducing changes of different types, i.e., to the physiological, biochemical, morphological, and molecular characteristics. Many traits besides yield are significantly influenced by drought stress. Early vigour of seedlings has also been reported as a beneficial trait that contributes to weed control, and water use efficiency and is likely to contribute to yield under certain environments, the reports were confirmed in field pea (*Pisum sativum* L.) which is represented by shoot biomass and seedling length^42^. In our study tolerant lines having higher DTIs were showing higher vigour values than the susceptible lines. LM22, had the highest vigour among all the lines. Similarly, chlorophyll content has been known as an index for evaluation of the source^43^, therefore decrease in the chlorophyll content can be considered as a yield-limiting factor in drought stress conditions. There are reports that resistant genotypes of wheat and corn had higher chlorophyll content than sensitive genotypes under oxidative stress^44^. A decrease in root parameters with increasing PEG concentration has been reported in tomato^45^, lentil^46^ and maize^47^. Root length was increased in the case of tolerant genotypes. The findings endorsed the fact that plants respond to drought by stimulating or maintaining root growth while reducing shoot growth^48^. Drought-tolerant tropical maize inbred lines have greater rooting depth than the sensitive lines^49^. The benefit of a deep and proliferative root system for drought tolerance has been reported in various crops including rice^50^, maize^51^, barley^52^ and wheat^53^. The box and whisker charts reported substantial variations between accessions and treatments in an experiment-II, as indicated by the lower and upper limits of the boxplots for root traits. Reports are found for various shoot and root biomass traits, germination and abnormal seedling percentage under two treatments of PEG 6000 (10% and 15%) in maize. The mean of the measured traits showed successive reductions as the PEG concentration increased from 10 to 15% within 16 days after sowing^54^ which, therefore, endorses our results of successive reduction in biomass with increasing content of osmolyte.

The plant vascular system is responsible for water and nutrient uptake, any type of changes in these functional processes are due to changes in vasculature in response to any stress, therefore it is considered imperative to study^55^. Anatomical alterations in the secondary tissues like xylem walls, metaxylem, the number and diameter of xylem and metaxylem, and endodermis cell wall have also been reported in rice^56^ and in maize^57^. for normal and water-deficient conditions Authors reported significant changes in the number of metaxylem vessels, cortex and xylem thickness and thinning of protoderm, endodermis in maize seedlings under normal and water-deficient conditions. The drought imposed through osmolyte (PEG) caused significant changes in number and diameter of the xylem and metaxylem in susceptible line. However, in LM22, no reduction in number of the secondary tissues was found and the decrease in diameter was also minimal. Under water-stressed condition, the number of meta xylem and xylem should increase to improve the hydraulic conductivity in the plant, as one of the adaptive traits. A smaller xylem vessel area as apparent from the reduction in the diameter of the root can reduce axial hydraulic conductivity. This can be advantageous under water-limited conditions by conserving soil water throughout the growing season, a strategy known as “water banking”^58^. Reduced hydraulic conductivity may prevent desiccation of the growing root tip and surrounding soil throughout the growing season, as well as moderate shoot water stress by increasing shoot water use efficiency. But in our study tolerant inbred line showed a reduction in diameter which was significant from the control as a response to the decreased water content, whereas in the case of the susceptible line, though the diameter of vessels was more reduced than tolerant line but the significant decrease in vessel number especially xylem will hamper the hydraulic conductivity needed to suffice shoot mass under drought. However some studies have reported an increase in, metaxylem number in maize which improves root hydraulic conductivity, while at the same time the total cortical area was reduced, which decreased the metabolic cost of accessing water in deeper soil domains^59^.

## Conclusion

The hydroponic-based drought stress screening technique is rapid, low cost and simple, as the whole process took only a few weeks to identify tolerant and susceptible maize genotypes. According to the findings of our experiments, root length and root biomass are related to drought tolerance, and these traits can be employed as selection criteria for the identification of WDS tolerant lines even at the seedling stage. The existence of inherent variability, mandatory for effective selection, was ensured through a large and diverse genetic stock. The authentication of a selectant group of lines with simulated WDS conditions led to the selection of validated candidate donor inbreds. It is recommended that the inherent plasticity response of genotypes to WDS needs to be captured and utilized for selection against complex traits. The selection based on anatomical changes under stressed and non-stressed conditions will pave a way for refined phenotyping at an early stage to select and breed for WDS tolerance in maize.

## Materials and methods

### Material

Seventy-one diverse maize inbred lines (supplementary Table 1) of diverse origin maintained at Punjab Agricultural University, Ludhiana; Maize section developed through continuous selfing/sib mating, were grown under a hydroponic system in poly house facility in 2021.

### Experiment I

#### Growth conditions and experimental design

The medium for germination was coco-coir as it has neutral pH, better aeration and also acts as a fair absorbent to retain the moisture. Seeds of each inbred lines were washed with sodium hypochlorite and raised in seedling trays for 15 days. Seedlings were transferred to plastic containers/meshed pots with clay balls as supporting media and placed in pipes. The seedlings raised using optimal water conditions were referred to as control, whereas the seedlings raised using osmolyte [different concentrations of (PEG6000)] were referred to as water deficient stress (WDS) treatment. The test material was evaluated in randomized complete block design comprising two replications each for control and treatment. Treatment comprised induction of osmotic stress using PEG6000 (each pipe sustaining 20 samples) with tanks (100 l each) supplying nutrient solution. The experiment was performed in a climate-controlled poly house with a 16 h light/8 h dark photoperiod, a temperature cycle of 25 °C/18 °C (day/night), and 65% relative humidity. The nutrient solution was Hogland’s media in each tank (100 l tank). The nutrient solution was aerated continuously and renewed every week and the pH was maintained by adding 1 M H_3_PO_4_. Distilled water was added regularly to maintain the volume. 30 day-old seedlings were given the treatment of 10% PEG6000^60^ to induce the osmotic stress.

The data for vigour was based on a scale from 0 to 4^61^ viz., 0 (representing dead), 1 (very poor), 2 (poor health), 3 (reduced vigour but with browning of leaf tips), 4 (deep green leaves with no wilting or chlorosis) and chlorophyll was measured at five leaf stage in µmol/m^2^ using MC-100 Chlorophyll content meter Apogee instrument. The crop was harvested 60 days after sowing (DAS).

#### Evaluation of root and shoot traits

At harvest, seedlings were carefully taken from the growing medium avoiding any breakage to the roots. The shoots of each plant were separated by cutting at the base of the stem and washed thoroughly to remove any impurities. After removing shoots, roots were laid on a flat surface and stretched to measure their length (from the base of the stem to the tip of the root system) as an estimate of rooting depth. The fresh weight of root and shoot of each seedling was recorded using manually by weighing balance. Then roots were scanned for image analysis with a root scanner (Biovis PSM- R2000) for different parameters viz. Root projection area/surface area (RPA) representing coverage of roots, average root diameter(ARD), estimated total root volume (TRV), root tips and forks (representing laterals) and total root length (TRL). The dry weight for root and shoot was recorded after 72 h of oven drying.

#### Grouping of lines based on seedling traits

Principal component analysis was performed in R: version 0.4.5 and conducted on data set of 13 seedling traits The criteria followed for selecting the principal components were based on Eigen values^62^ and the Eigen values above unity indicated that the evaluated principal component is reliable^63^. Based on reduced unrelated 10 traits, clustering was performed through heatmap (clustelVis; pheatmap R package version 0.7.7) and ranking of genotypes for their response to moisture stress was done based on drought tolerance indices (DTI) computed for seedling traits. DTI was worked out for the important traits using formula^64^

$${\text{Drought Tolerence Index}} = \frac{{YP \times YS}}{{\hat{Y}p^{2} }}$$

*YP* = value under control, *YS* = value under stressed condition, *Ŷp*^2^ = square of mean under control.

### Experiment II

Based on DTIs 20 lines (Table5) with differential response to drought tolerance ranging from most tolerant to the least were taken from 71 inbreds and selected for further extensive pot studies. The experiment comprise six sets: two replicates of control and two replicates of each of two treatments viz*.,* 15% and 20% PEG6000 for creating water stress. The seeds were directly sown in plastic transparent glasses with the capacity of (250 cm^3^) to avoid the root injuries by pulling and filled with field soil (clay loam, for mimicking the field conditions). Each inbred was replicated in three pots The experiment was divided into six sets representing two replications for control, 15% PEG6000 (T1) and 20% PEG6000 (T2). Hogland’s media for nutrition was given at weekly intervals. The experiment was conducted in natural conditions under Randomised complete Block Design. Both the concentrations of PEG6000 were given 10 days after germination (DAG). The solution of the osmolyte was replenished every day for twenty consecutive days in both treatments whereas distilled water was used for the control representing well-watered (WW) conditions. After 20 days, the seedlings were harvested and used for recording various traits for roots viz*.,* RSA, TRV, TRL and ARD using image analysis and for shoot including seedling length; shoot length SL and maximum  root length (MRL) as mentioned previously were recorded.

### Experiment III

#### Root anatomical studies in extreme inbreds for WUE

Based on DTI values for major root and shoot traits, two extreme maize inbred lines for drought viz., LM 22 (most tolerant) and CML 494 (most susceptible) were selected for root anatomical studies, at high resolution using SEM (JSM-7610FPlus:JOEL).

#### Sample preparation

**Figure 6:**
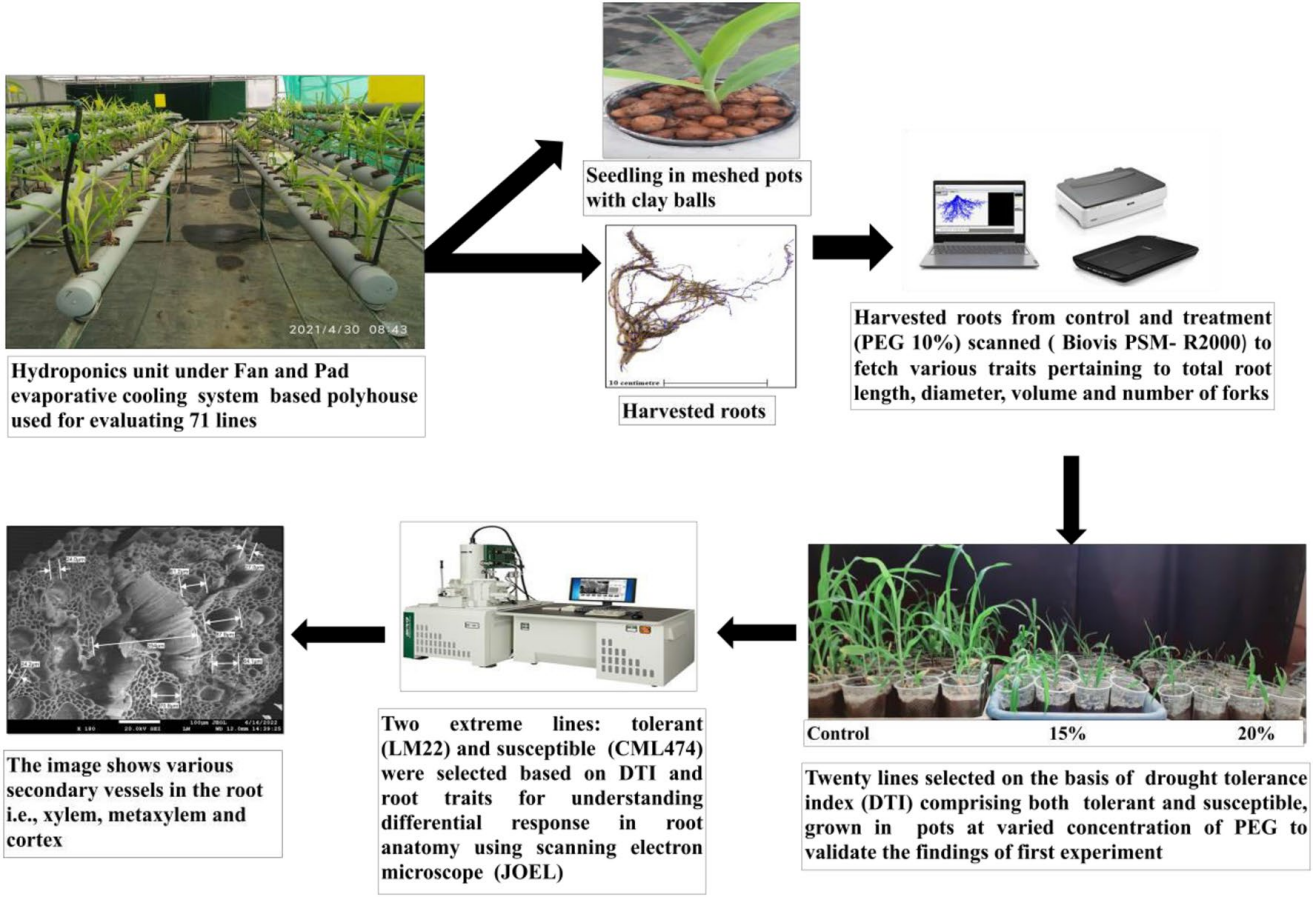
Representation of whole experiment in the pictorial way.

The seeds of each maize line were sown in plastic pots of 250 cm^3^ capacities and filled with autoclaved sand. The experiment comprised a set of two pots for each line in each replicate in well-watered as control and a similar set of pots for treatment with osmolyte (20% PEG6000). Hogland’s solution was used for nutrition for each set. The treatment of the PEG6000 (20%) was started seven days after germination (DAG). The experiment was terminated after 40 DAS under the pot, as the growth was slow. The seedlings were harvested and thoroughly washed. The roots were cut from base of the stem and wrapped in tissue paper. The sample preparation was done in the Nanotechnology laboratory, PAU. The roots were cut in 2 mm size. Chemical fixation of root samples was done with 40 ml of 2.5% glutaraldehyde (for cross-linking of proteins and seizing the existing stage) in 10 ml of 2 M Sodium cocadylate (final volume 100 ml). The solution was filled in vials till the specimen was completely immersed and kept overnight at 6–8 °C. After 24 h, three washings of Sodium cocadylate with an interval of 15 min were done. Samples were immersed in 2% buffered Osmium tetraoxide (to prevent the extraction of lipid membrane) for 25 min at 6–8 °C, then repeated the above step of washing with Sodium cocadylate. Standard dehydration was performed with a graded series of organic solvents (ethanol: 30%, 50%, 70% and 100%) for 15 min each to gradually remove water without causing specimen shrinkage. followed by critical point drying (CPD)^65^. The samples were transferred to a critical point dryer to allow solvent to evaporate from the specimen surface. Then the specimens were mounted on metal stubs using carbon discs. To increase the conductivity, sputtering with gold was done for eight minutes and then samples were scanned under SEM (JSM-7610FPlus:JOEL.) at different magnifications and scale bars. The pictorial representation of whole experiment is given as Fig. 6.

### We confirm that all methods were performed in accordance with the relevant guidelines and regulations

#### Data collection and analysis

Phenotypic data for various root and shoot traits were recorded. Analysis of variance (ANOVA) was conducted to characterize the variation for the seedling in OPSTAT^66^. CLUSTER heatmap (clustelVis; pheatmap R package version 0.7.7) was used for multivariate analysis, PCA was performed in R package (Version 0.4.5). Box and whisker charts (Ms –Excel 2019) were used to decipher the variation in germplasm for root traits.

## Supplementary Information

Supplementary Information.

## Data Availability

All the data analysed during the study are included in published article.
