# Supplementary material for: Root plasticity: an effective selection technique for identification of drought tolerant maize (Zea mays L.) inbred lines
Source: Sci Rep. 2023 Apr 4;13:5501. doi: 10.1038/s41598-023-31523-w (PMC10073119; doi:10.1038/s41598-023-31523-w)
Supplement: Supplementary file 1 — Supplementary Information. [file 41598_2023_31523_MOESM1_ESM.docx]

**Table S1**: List of 71 inbred lines used under investigation

| **S.N** | **Lines** | **Pedigree** | **Breeding line** |
| --- | --- | --- | --- |
| 1 | LM5 | Tux Pool C2 IC2-5-1-1-2-2-2-2-3-1f | PAU |
| 2 | LM6 | MS Pool C2 IC2-5-1-2-1-1-2-1-1f | PAU |
| 3 | LM11 | Suwan 1-26-1-1-1-1-1-1-1-1-1-f | PAU |
| 4 | LM12 | J 54 Mo 17-21-2-3-2-2-1-1-2-1-2-1-f | PAU |
| 5 | LM13 | JCY 3-7-1-1-1-2-3-f | PAU |
| 6 | LM14 | CA 00 310-1-1-1-3-1-f | PAU |
| 7 | LM15 | JS2 x J3022]HS 80 -1-1-1-1-#-FS3-FS2-#-#-#-selfb-#-# | PAU |
| 8 | LM16 | [JS 4-30..x(Tarun x MS 1)-yellow-63-1g..]-12-4-1-4-2-1-selfb-### | PAU |
| 9 | LM17 | (Partap 69-2-1-2-1-1-1-1-1-1-1-……( LMP-1)-1 | PAU |
| 10 | CML387 | [EV7992#/EV8449-SR]C1[F.sub.2]-334-1(OSU8i -1-1-B-B-4-B*3 | CIMMYT |
| 11 | CML444 | P43C9-1-1-1-1-1-BBBB | CIMMYT |
| 12 | CM452 | Ac8328BNC8-161-1-1-1-8BBBBBB | CIMMYT |
| 13 | CML494 | LAPOSTASEQ-C4-F7-1-2-2-2-2-B | CIMMYT |
| 14 | CML511 | [CML389/CML176]‐B‐29‐2‐2‐B*5 | CIMMYT |
| 15 | CML533 | SA4-C4-FS19-3-2-2-2-3-4-3-B | CIMMYT |
| 16 | CML539 | MAS(MSR/CML312)-117-2-2-1-B | CIMMYT |
| 17 | CML545 | (CML312/CML445//(TUXPSEQ-C1-F2/P49SR)-F2-45-3-2-1-B)-1-2-1-1-2-B | CIMMYT |
| 18 | CML551 | P27FRRS-C1-18-B-1-1-B | CIMMYT |
| 19 | CML561 | (G9A-C7/SR)/G9A-C7)/G9A-C7)-FS59-4-1-2-1-1-1-B | CIMMYT |
| 20 | CML574 | (LINESP27&G17/LINESNORBSSS)-53-2-2-1-B-1-B*8 | CIMMYT |
| 21 | CML575 | (CML451/CLRCW29)-B-38-2-B-1-B*7-1-B-B | CIMMYT |
| 22 | CML576 | (CLFAWW11/CML494)-B-24-2-2-B-B-1-B-8-B-B | CIMMYT |
| 23 | CML577 | (CML454/CML451)-B-16-1-1-2-1-B-7-B-B | CIMMYT |
| 24 | CML546 | (CML312/CML445//(TUXPSEQ-C1-F2/P49SR)-F2-45-3-2-1-B)-1-2-1-1-2-B | CIMMYT |
| 25 | CML579 | WLC-Y-7-2-3-3-1-1-B-B-# | CIMMYT |
| 26 | CML504 | [COMPE2/P43SR//COMPE2]F#‐20‐1‐1‐B‐1‐BB‐6‐BB | CIMMYT |
| 27 | LM20 | CML 25-1-1-1: P26-FS37-1-4-B | PAU |
| 28 | LM22 | SSPool#-6-4-3-2-1-1### | PAU |
| 29 | LM23 | SE 569 | PAU |
| 30 | LM24 | (CML 32XCML25)##-2-6-1-1-1-1-## | PAU |
| 31 | LM25 | SE 563 | PAU |
| 32 | LM26 | JCY 45-1-1-1-1-1-1-1 | PAU |
| 33 | PML7 | (MS C2 IC2-3-2…x MBR plot 102-6-1…)#-1-1-1-1-1-1-1-1-1-1-1-1- *f* | PAU |
| 34 | PML22 | LM 6 x CM L 72-1-2…)-b-b-12-1-1-1-1-1-1-1-1- *f* | PAU |
| 35 | PML24 | OP ear-4-20-1-1-2-1-1-1-1-1-1-1-1-1- *f* | PAU |
| 36 | PML25 | Tux C2 IC3-7-1-1-2-1-1-1-1-1-1-1-1-1- *f* | PAU |
| 37 | PML45 | (Tux 162 x Tux C2 IC2-5-1..)-1-1-1-1-5-1-1-1-1-1-1-1- *f* | PAU |
| 38 | PML46 | (Tux 162. Tux C2 IC2-5-1…)-4-1-1-1-4-(OP)-1-1-1-1-1-1-1-1- *f* | PAU |
| 39 | PML48 | (Tux 162 x Tux C2 IC2-5-1…)-37-1-2-2-1-1-1-1-1-1- *f* | PAU |
| 40 | PML49 | (Tux 162… x Tux C2 IC2-5-1…)-4-1-1-1-6-1-1-1-1-1-1-1- *f* | PAU |
| 41 | PML51 | (Tux 162.LM 5-6-1..x Sw1 155086-4-3)#-1-1-2-1-1-1-1-1-1-1-1-1-1 | PAU |
| 42 | PML52 | (Tux 162.LM 5-6-1..x Sw1 155086-4-3)#-1-1-2-1-1-1-1-1-1-1-1-3-1 | PAU |
| 43 | PML53 | (Tux 162.LM 5-6-1..x Sw1 155086-4-3)#-1-1-2-1-1-1-1-1-1-1-1-4-1 | PAU |
| 44 | PML55 | (Tux C2 IC2-3-1…x Sw1-155086-8-4…)#-1-1-2-1-1-1-1-1-1-1-1 | PAU |
| 45 | PML61 | (Tux C2 IC2-15-2..x Tux C2 IC2-12…)-26-1-1-1-4-1-1-1-1-1-1- *f* | PAU |
| 46 | PML67 | (P1 x EC 2532-40-2..x P1 x EC 255239)-5-4-1-1-1-1-1-1-1-1-1- *f* | PAU |
| 47 | PML73 | (J 54 Mo 17-21…x CIMMYT MBR plot 102-6-1…)-6-1-2-1-1-1-1-1-1-2-1-1 | PAU |
| 48 | PML76 | J 54 Mo 17-21…x CM 71)#-b-b-1-1-1-1-1-1-*f* | PAU |
| 49 | PML86 | Pioneer-13-1-1-3-1-1-1-1-1-1-1-1-1-fx LM 13#-1------f | PAU |
| 50 | PML95 | (Pio 30 A 97 468654)-b-1-1-1-1-1-1-1-f x LM 13#-1------f | PAU |
| 51 | PML97 | (Pioneer Hyb LEP Long Ear) -27-2-1-1-1-3-1-1-1-1-1-1-1-1-2-1-1-f x LM 13#-1------f | PAU |
| 52 | PML98 | CML 20/LM 13-1-1-1-3-4 xLM 13 # -1-2-1-1---- *f* | PAU |
| 53 | PML104 | CML 430/LM13-2-1-1x LM 14# -1-1-1-1-1- *f* | PAU |
| 54 | PML115 | DK 999 (EC 468659-) b-1-1-1-4-1-1-1 | PAU |
| 55 | PML118 | PAC 9757 x-b-1-1-1-f | PAU |
| 56 | PML145 | NECH 109 –b-1-1-1-1-1-1-1-1-13#-1------f | PAU |
| 57 | PML150 | (SE 503 x SE 513)-S8-3-1-1-1--13#-1------f | PAU |
| 58 | PML156 | PT 963052-B-B-B-B-1-1-1-1-1--13#-1------f | PAU |
| 59 | PML161 | C 919 Cargill EC 468658-b-1-1-1-1-1-1-1-1-13#-1------f | PAU |
| 60 | PML166 | CL03618XCML287)-b-1-1-1-1-1-1-1-1-1-1-f | PAU |
| 61 | PML170 | (CML 285 x CL 02410)-b-3-1-1-1-1-1-1-1-1-1-1 | PAU |
| 62 | PML187 | EVT13 J & K S 9528 (Thailand) -b-1-1-1-1-1-1-1-13#-1------f | PAU |
| 63 | PML158 | C 919 Cargill EC 468655-b-1-1-1-1-1-1-1-1 | PAU |
| 64 | PML207 | DMR 201 P102 E2-1-B-1—f/LM 13-#-1-1-2-1-1-*f* | PAU |
| 65 | PML250 | HS- 2787-2-1-1-4----13#-1------f | PAU |
| 66 | PML270 | HS-2816-1-1-(2)-1-2-1----13#-1------f | PAU |
| 67 | PML276 | HS-2818-1-1-(2)-1-2-1-1 | PAU |
| 68 | PML296 | WN 6143-1-1-1-1-1-1--13#-1------f | PAU |
| 69 | PML365 | JCY3 -7-1-2-1-b-2-1-2-1-1-1-1 | PAU |
| 70 | PML373 | LM 13 A’-1-1 | PAU |
| 71 | PML375 | LM 13 C-1-1 | PAU |

PAU: Punjab Agricultural University, Ludhiana, Punjab
